# Professional development of medical students – piloting a longitudinal curriculum at Jena University Hospital (LongProf)

**DOI:** 10.3205/zma001699

**Published:** 2024-09-16

**Authors:** Konrad Schmidt, Katharina Siller, Jens Rißmann, Marie Andlauer, Jana Feustel, Friederike Klein, Inga Petruschke, Sven Schulz

**Affiliations:** 1 Charité Campus Mitte, Institute of General Practice and Family Medicine, Berlin, Germany; 2 University Hospital Jena, Institute of General Practice and Family Medicine, Jena, Germany; 3 Asklepios Fachklinikum Stadtroda, Stadtroda, Germany; 4 Jena University Hospital, Department of Internal Medicine I, Jena, Germany

**Keywords:** curriculum, medical professionalism, mentoring, medical studies, relationship-based learning, professional identity formation, situated learning

## Abstract

**Background:**

Professionalism is an important prerequisite for the quality of medical care with specific competencies anchored in the National Competence-Based Learning Objectives Catalogue Medicine 2.0. To date, there are hardly any explicit teaching formats at German universities to achieve these. A longitudinal curriculum for the development of medical professionalism (LongProf) has now been developed, implemented and evaluated at Jena University Hospital.

**Methods:**

The target group of the four-semester-curriculum were medical students from the fifth semester onwards. After a nine-month conception phase, a total of nine courses (6 teaching units each) took place from the winter semester 2021/22. Students also had the opportunity to interact with experienced doctors in mentoring sessions. The courses were evaluated by the participating students (n=23) in terms of acceptance and individually perceived professional development through quantitative surveys and qualitative focus group interviews.

**Results:**

The qualitative and quantitative evaluation revealed mostly positive feedback (mean >7/9). Students stated that the courses had provided them with lasting support in developing their own medical professionalism and in coping with the demands of their studies. The personal and long-term relationship building between students and teachers was considered particularly helpful.

**Discussion and conclusion:**

A multi-semester curriculum opens up ways for implementing the development of medical professionalism in medical studies. A trusting relationship between students and teachers, made possible by the longitudinal structure, is seen as conducive to the development of an individual medical identity. The curriculum is a useful complement to regular medical studies.

## Zusammenfassung

**Hintergrund:**

Professionalität ist als wichtige Voraussetzung für die ärztliche Behandlungsqualität mit spezifischen Kompetenzen im Nationalen Kompetenzbasierten Lernzielkatalog Medizin 2.0 verankert. Um diese zu erreichen, existieren an deutschen Hochschulen bislang kaum explizite Lehrformate. Am Universitätsklinikum Jena wurde nun ein longitudinales Curriculum zur ärztlichen Professionalitätsentwicklung (LongProf) entwickelt, implementiert und evaluiert.

**Methodik:**

Zielgruppe des über vier Semester stattfindenden Curriculums waren Medizinstudierende ab dem fünften Fachsemester. Nach einer neun-monatigen Konzeptionsphase fanden ab dem Wintersemester 2021/22 insgesamt neun Veranstaltungen statt, mit jeweils sechs Unterrichtseinheiten. Zusätzlich bestand für die Studierenden die Möglichkeit, mit erfahrenen Ärztinnen und Ärzten über mentoring-Angebote in Austausch zu treten. Die Lehrveranstaltungen wurden durch die teilnehmenden Studierenden (n=23) hinsichtlich Akzeptanz und individuell wahrgenommener Professionalitätsentwicklung durch quantititative Befragungen und qualitative Fokusgruppeninterviews evaluiert.

**Ergebnisse:**

Die qualitative und quantitative Evaluation ergab mehrheitlich positive Rückmeldungen (MW>7/9). Die Studierenden gaben an, durch die Lehrveranstaltungen in der Entwicklung ihrer eigenen ärztlichen Professionalität und bei Bewältigung ihrer Studienanforderungen nachhaltig unterstützt worden zu sein. Als besonders hilfreich wurde hierbei der persönliche und langfristige Beziehungsaufbau zwischen Studierenden und Lehrenden empfunden.

**Diskussion und Schlussfolgerung:**

Ein semesterübergreifendes Curriculum eröffnet Wege zur Implementierung einer ärztlichen Professionalitätsentwicklung im Medizinstudium. Als förderlich für die Entwicklung einer eigenen ärztlichen Identität wird eine vertrauensvolle Beziehung zwischen Studierenden und Lehrenden empfunden, ermöglicht durch die longitudinale Struktur. Das Curriculum stellt eine sinnvolle Ergänzung des regulären Medizinstudiums dar.

## Introduction

As an essential component of the medical profession, professionalism goes far beyond the acquisition of medical skills and expertise. The internationally recognised “CanMEDS” model of the Royal College of Physicians and Surgeons of Canada describes professional physicians as possessing values such as high professional competence, integrity and a sense of responsibility, as well as a specifically trained reflexivity that is equally directed towards themselves, the medical profession, patients and the social environment [1].

According to Irby et al. [2], the formation of a professional identity is subject not only to clinical and non-clinical experiences, but also to individual expectations, beliefs, commitments and environmental factors [3]. Critical factors are the student's own understanding of professional and ethical behaviour, reflective action and supportive relationships through which medical students develop personally and professionally as physicians [4], [5].

The International Charter on Medical Professionalism emphasises a commitment to the three fundamental principles of “patient welfare, patient autonomy and social justice” [6]. The commitment to competence, morality, altruism and the promotion of the common good forms the basis of a social contract that grants the health professions in particular a monopoly on the use of their knowledge base, the right to extensive autonomy in practice and the privilege of self-governance [7].

The development of professional identity begins with the decision to enter the profession and is a process that continues throughout the career [8]. There is a consensus in international medical education that medical professionalism and its various aspects should be explicitly taught [9], [10].

### General needs assessment

Longitudinal curricula exist in German-speaking countries, for example to teach communicative [11] or primary care skills [12], but with a few exceptions [8] not to develop professionalism. This may be due to the lack of a standardised definition of “medical professionalism”. Although the National Competency-Based Learning Objectives Catalogue Medicine (NKLM 2.0) already includes competency-based learning objectives for the development of professionalism [https://nklm.de/zend/menu], existing curricula often follow a tradition of teaching knowledge and skills only [13]. In addition, many standard courses are characterised by a high degree of specialisation and frequent changes in teaching staff. Moreover, large numbers of students lead to an anonymous teaching and learning environment; personal relationships between students and teachers are rare. However, relationship-oriented learning appears to be essential [14] to encourage future doctors to integrate values and attitudes into their own professional identity.

Based on this initial situation, the project “LongProf: Longitudinal Curriculum of Medical Professionalism” was initiated at Jena University Hospital in 2021. This article presents the development and piloting of the curriculum with evaluation results and the experiences of the working group.

## Project description

The project was funded by the Strategy and Innovation Fund of the Federal State of Thuringia between 2021 and 2023. The core of the working group consisted of two psychologists and five doctors from the Institute of General Medicine and the Department of Internal Medicine I at Jena University Hospital. The development of the curriculum took nine months and was adapted from Kern [15].

### Needs assessment of the learners

At Jena University Hospital, there have not yet been any courses that explicitly address the development of medical professionalism. In addition, the working group identified the tension between economisation and patient welfare [16] and the topic of “professional self-care” for sustainable student and physician health [17] as relevant to medical education.

### Definition of overarching and specific learning objectives

Based on an exploratory literature review, the learning objectives of LongProf are based on the above-mentioned CanMEDS roles [1], the International Charter on Medical Professionalism [6] and the NKLM 2.0 [https://nklm.de/zend/menu] (see attachment 1 ).

### Teaching and learning methods

**Figure 1:**
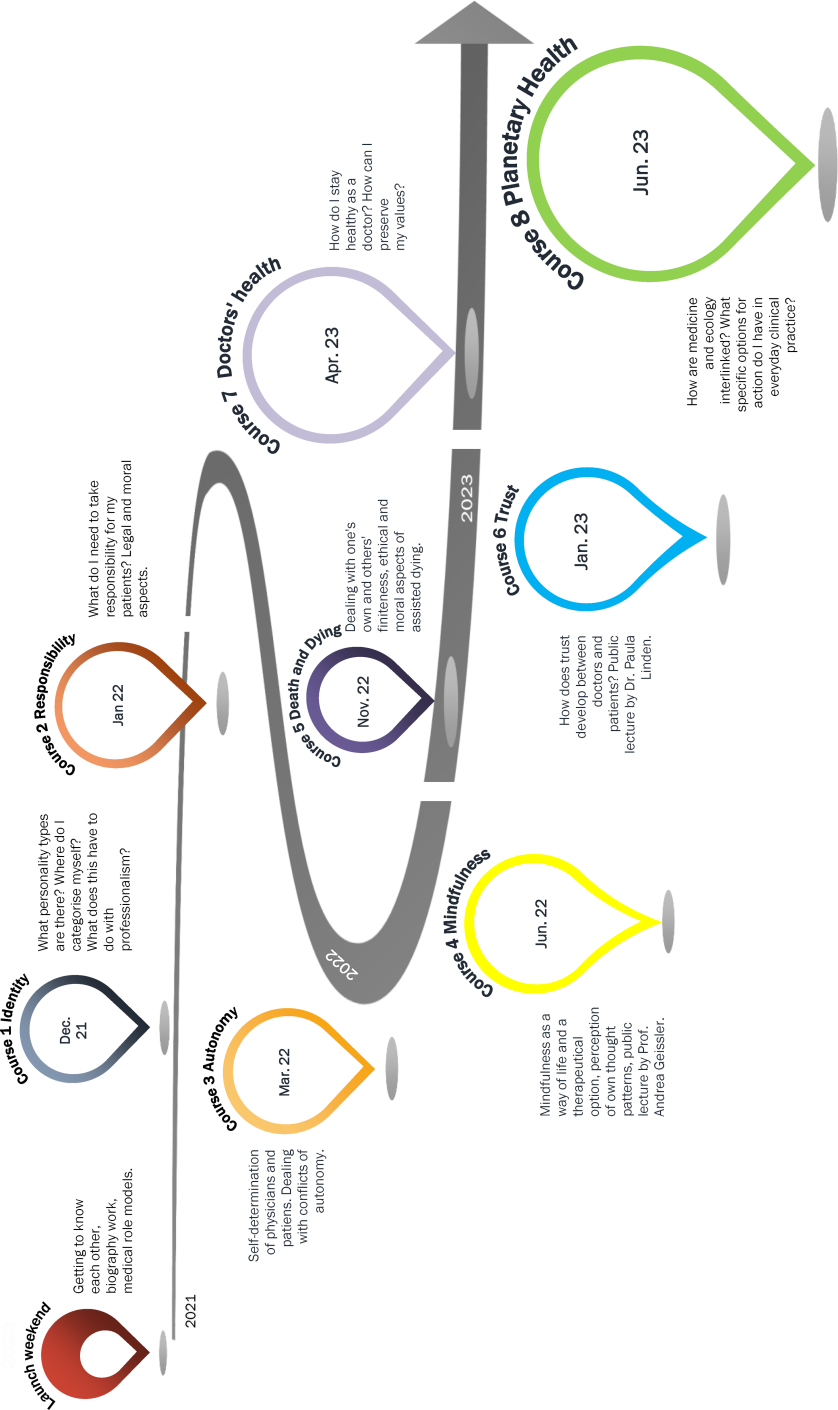
Course structure of the LongProf curriculum

Based on the above theoretical background, the learning objectives and the faculty’s own emphases, the curriculum was structured around the themes of “identity”, “responsibility”, “autonomy”, “mindfulness”, “death and dying”, “trust”, “physician health” and “planetary health” (see figure 1 (Fig. 1)).

In addition to the transfer of knowledge, an integral part of each course was always concrete personal experience, which could be gained, for example, in role plays or by working on case vignettes [7]. These experiences were then reflected on by the students in a protected environment [7], [13], [18], [19]. At the end of each course, there was an additional opportunity for the students to discuss practice-relevant aspects they may encounter later in their careers with a doctor in training, in order to work out the concrete relevance of the reflection results for the transition from studying to working life.

**Figure 2:**
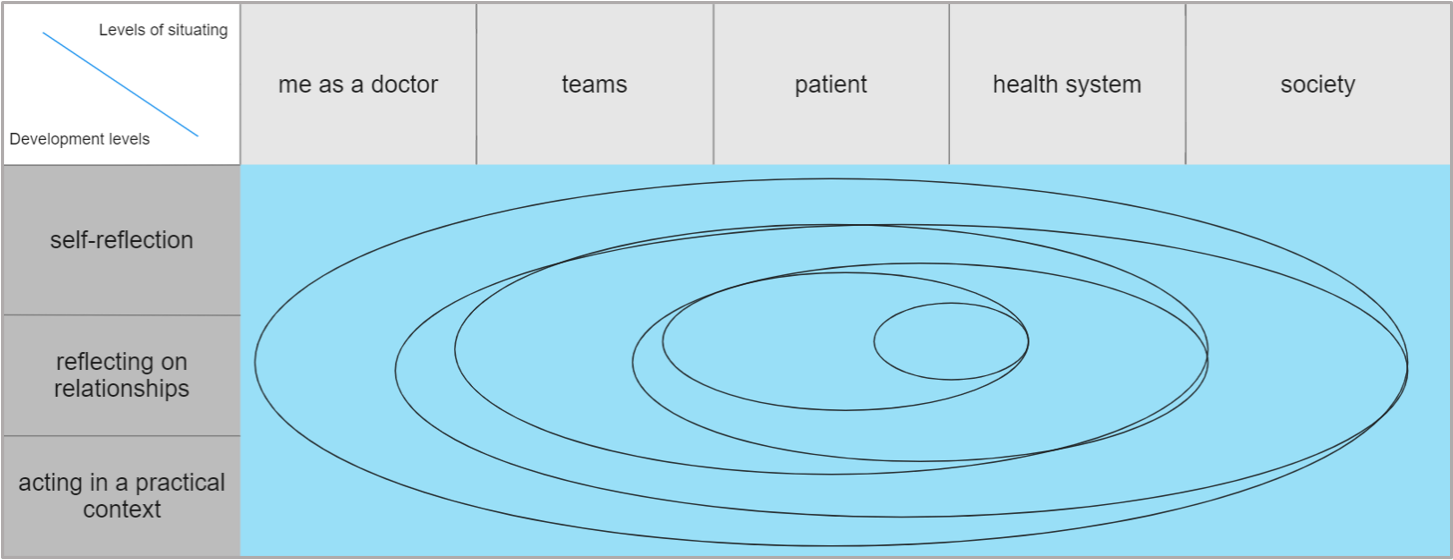
Situational model according to Rißmann as a framework concept for the LongProf curriculum [23]

A “situational model” was developed as a framework for methodological implementation (see figure 2 (Fig. 2)). It was assumed that professional identity development can be described as situated and reflective engagement in medical contexts. “Situated” learning [20], [21] is contextualised and sees learning not as a purely cognitive process, but as an interplay of thinking, feeling and acting in specific environments and situations. “Reflective” emphasises the understanding of contexts and relationships within the learning process and includes all experiences, ideas, emotions and cognitive thought processes [22].

In our approach to professional identity development, students should consciously engage with the five situational levels of doctor, team, patient, healthcare system and society in order to develop responsible action and active relationship building. The selection of these five situational levels is based on the practical experience of the faculty involved and does not claim to be exhaustive.

The three levels of development describe three interdependent dimensions of the learning psychology of medical students:

1. *Self-reflection of experiences *related to the above situational levels forms the basis for understanding complex contexts.
2. *Reflection on relationships* shifts the focus from the self to the direct counterpart of social systems for shaping professional relationships.
3. Finally, *action in practical contexts *transfers the processes of reflection from the protected space (of study) to practical and responsible action in real situations of everyday professional life.

From a methodological point of view, the iterative construction and deconstruction of students' experiences at the above-mentioned levels can develop professional beliefs, values and behaviours and thus contribute to the formation of a professional identity [3]. Other levels can be added and differentiated as needed.

### Implementation of the curriculum

The curriculum was piloted from the winter semester 2021/22 with students from the 5^th^ and 7^th^ semesters, who had volunteered from a semester size of around 250 students each. The curriculum ended in the summer semester of 2023. The curriculum was already advertised to students in the then 4^th^ semester in the summer semester of 2021 using a video and an information event. A survey in this semester (N=112) showed that 14% would participate in such a curriculum.

The cohort was divided into two groups, each of which was accompanied by a “teacher-tandem” consisting of a psychologist and a doctor. All courses were held in person in compliance with the current hygiene rules during the SARS-Cov2-pandemic.

As a launch event, a weekend seminar was held in a rural hostel outside Jena in the winter semester 2021/22. The content included intensive biographical processing of the participants' own career aspirations and a “world café” to exchange ideas with representatives of various medical disciplines as potential role models (see also attachment 1 ).

Subsequently, two courses of six teaching units (TU, 45 min.) each took place each semester - both on university premises and in locations specific to the course topics, e.g., in the prayer room of a church (“death and dying”) or in a circus tent (“autonomy”). The basic structure of each course includes an informal exchange on current student issues, frontal knowledge transfer (approximately one TU), an experimental self-experience, space for subsequent reflection and exchange on practical application (see table 1 (Tab. 1) or described in detail in the curriculum manual) [23].

Students were also able to take advantage of fortnightly mentoring sessions offered by individual teachers on a rotating basis. This framework was designed to facilitate a constructive and confidential exchange of experiences between a professional and the students, even outside the courses. In addition, students received a fortnightly “impulse letter” by email, in which a teacher shared medical topics or inspiring personal experiences. An accompanying journal was also developed for the students, with further suggestions and impulses in the form of questions and tasks, as well as space for their own notes, as a personal space for reflection to broaden their own perspectives and to come closer to their own professional medical identity.

In addition to the internal curriculum courses, three public university lectures were offered with external speakers on topics related to the LongProf curriculum (see attachment 1 ).

The curriculum was extended to include student initiatives through the so-called “free offers”. This systemic-dynamic approach created an intersection between professionalism and individual experiences of students and teachers. In addition to medical topics, personal experiences could be incorporated into identity development, for example in archery or cross-country skiing.

## Evaluation

The aim of the accompanying quantitative and qualitative evaluation was to record the students’ individual perception of their professional development and their acceptance of the curriculum [24]. There were no optional or compulsory assessments for students. The design, implementation and analysis of the evaluation were largely carried out within the project. The qualitative evaluation was carried out by an external member of staff from the institute of general practice in Jena.

A quantitative questionnaire, based on the university project “Lehrevaluation” (ULe) [25], collected socio-demographic information and assessed didactics, practical relevance, relationship to participants and commitment of the teaching staff separately for each course, using a Likert scale from 1 to 9 (1=“strongly disagree” to 9=“strongly agree”). The questionnaires were adapted for each course and sent out after each course via the online platform “SoSci Survey” [https://www.soscisurvey.de] (see table 1 (Tab. 1) and attachment 2 ). The use of the journal and the external offerings were not evaluated separately.

The evaluation was carried out descriptively by calculating means and standard deviations. Cronbach’s alpha was calculated as a measure of internal consistency to determine the reliability of individual items for the evaluation of events.

Following a mixed-methods design [23], a qualitative, guideline-based group interview was also conducted after the first semester with two students from each group (see attachment 2 , interview guidelines). The discussions were recorded, pseudonymised and transcribed using software (f4, Dr. Dresing & Pehl GmbH). The extraction of main and secondary categories was carried out independently by two members of staff using Mayring’s qualitative content analysis [26].

## Results

A total of 21 students participated in the courses in the winter semester 2021/2022, mainly from the 5^th^ semester, about a quarter of them male. In the summer semester of 2022, an additional four students joined, and in the winter semester of 2022/23, a further eight students joined, partly through the faculty's internal advertisement, and partly through personal contacts.

Both the courses and the teachers were rated positively over time, with mean scores >7 out of a maximum of 9 points (see table 1 (Tab. 1)). In particular, the development of medical professionalism – the main objective of the project – was promoted according to the participating students.

The average rating of the teaching staff was higher than that of the courses (see table 2 (Tab. 2)). In particular, according to the students, the teaching staff facilitated a cooperative and open-minded course atmosphere (mean=8.52; SD=0.91).

The acquisition of practice-relevant knowledge was rated nominally lowest by the students across all courses, and lowest for the “launch weekend” (mean=6.47; SD=1.41). Descriptively, a slight increase in this rating was observed over time. A breakdown of all results by question and course can be found in oder attachment 3 .

The reliability of the scales used was good to very good. The internal consistency of the questions on the courses was good (Cronbach’s alpha=.917), and that of the questions on the teaching staff was satisfactory (Cronbach’s alpha=.885). The evaluation methods used were not able to show any correlations between the development of the entire group.

The guideline-based group interviews revealed a high level of acceptance and appreciation of the curriculum (see table 3 (Tab. 3)). The majority of the students interviewed stated that they would like to see this offered in medical studies in the future. In particular, the strengthening of self-reflection was perceived as helpful for future professional activity. The relatively long duration of the courses (six TUs) had a positive effect on this. The participants perceived a strengthening of their ability to cope with their studies and reported that they were able to develop new approaches to problem solving and stress reduction. The participants felt that the teachers perceived them personally and as equals. Relationships between participants were also described as very supportive. 

The offer of accompanying mentoring was used a total of eleven times over three semesters with different teachers. According to the group interviews, the appointments were experienced as inspiring and provided a high level of reassurance. The female doctors in training played an important role for the students both professionally and as personal reference persons – particularly due to their similar age and the proximity in time to their medical studies.

## Discussion

With the development and implementation of the longitudinal curriculum “LongProf”, medical professionalism was explicitly addressed for the first time in medical studies at Jena University Hospital. In doing so, not only the NKLM 2.0 was considered, but also the wish of many medical students for an opportunity for self-reflection and the active practice of implicit professional competences [27].

With a planned maximum number of 40 participants and an initial response rate of less than 5%, the total number of participants was below expectations. The relatively low response rate can be explained by the heavy workload of medical students, their focus on courses directly relevant to examinations, the non-trivial communication of the implicit project objectives in short promotional formats, and possible uncertainties during the SARS-CoV2 pandemic.

The courses were rated positively by the majority of students. The teachers and their relationship with the students were rated even more positively, which was confirmed in the group interviews. In contrast to regular medical studies, the students felt that they were noticed on a personal level and benefited from a direct exchange of experiences with people in advanced stages of professional development. This suggests that the project objective of building positive relationships between teachers and students was achieved. The longitudinal structure of the curriculum enabled relationship-based learning for the development of an individual medical identity. The added value lies in the professionalism-focused verbal and non-verbal communication between teachers and learners, which enables the active acquisition of professional values and attitudes through explicit engagement with role models.

By comparison, the acquisition of practical skills was rated the lowest. This may be related to the fact that the overarching dimensions of professionalism in medical studies are perceived as less directly applicable than concrete recommendations for action (such as structured examination procedures or indications) that students are used to from other courses.

The focus of the curriculum described here was on the development of students’ professional identity and personality by addressing individual experiences and emotions. This required a deliberately longitudinal learning horizon in order to recognise and support implicit, ongoing developmental processes. Furthermore, the majority of students started the curriculum in the 5^th^ semester, when the relevance of such indirectly effective competences is presumably even less visible. This may have changed over the course of the curriculum, as indicated by the slight descriptive increase in the rating of practical relevance over time.

The future integration of an appropriate assessment format would be a conceivable way of giving students feedback on the competences they have acquired – in line with the learning objectives of NKLM 2.0. However, due to the multiple dimensions of professionalism, the development of such a format is by no means trivial and could also interfere with experience-based, situational learning.

The mentoring programme was only used irregularly by the students. On the one hand, this could be due to the fact that either the need for dialogue and/or the time budget available in the degree programme was less than expected. On the other hand, the students stated in the group interview that it would have been easier for them to use the programme if they had been given specific topics to discuss.

### Limitations

The quantitative findings are limited by the small number of cases involved in the project and the lack of a control group. Although the socio-demographic data of the participants were pseudonymised, it is conceivable that conclusions could be drawn about individuals in such a small sample. This means that socially desirable information cannot be excluded, which may have been exacerbated by the internal project implementation of the evaluation. The qualitative interviews, inductively conducted and analysed by an external staff member, are therefore an important complement to the quantitative data. If resources are available, a fully external evaluation should be considered in the future.

It was also noted that during the SARS-CoV2- pandemic, motivation to attend the face-to-face events (especially the launch weekend) was very high, which may have had a positive impact on the evaluation results.

The implementation of the courses was challenging in many ways: due to the compact semester schedule and the involvement of students from different semesters, finding dates for the courses proved to be organisationally complex. Although many students expressed interest, they decided not to participate in the LongProf curriculum due to scheduling conflicts and the additional time required.

Due to the small, possibly selected cohort, it is not possible to say whether the curriculum would be applicable and helpful to all student personalities in one semester.

### Perspective

The project could not be continued at Jena University Hospital due to the end of funding. The project leaders therefore decided to publish the methods and results freely [23]. This opportunity has already been used by colleagues at the Charité - Universitätsmedizin Berlin, where three cohorts started the adapted curriculum “LongProf Charité” in summer 2024 [28]. Here, the focus was on students in their first semester in order to support them throughout their entire study period. Due to the high level of self-motivation, a sufficient number of teachers from several departments volunteered to participate (Institute of general medicine/Clinic for psychiatry and psychotherapy). However, the authors recommend continuous support with recognition as a teaching achievement to ensure long-term sustainability.

Due to the lack of a standardised definition of medical professionalism, future teachers will have to constantly re-evaluate their individual views and attitudes in order to implement the curriculum. The handbook published by the authors [24] can be helpful in this regard, but it is by no means intended to be binding in its entirety.

Due to the limited feedback both in Jena and currently in Berlin in relation to the total semester size, compulsory participation does not appear appropriate at present, as the authors believe that the students’ self-motivation makes a decisive contribution to achieving the learning objectives.

## Conclusions

Medical professionalisation and personal development are rarely addressed so far in standard medical studies. The LongProf pilot project has demonstrated the feasibility and acceptance of a curriculum in this area. This opens up ways for a permanent implementation of the topic. The longitudinal nature of the project enabled a personal relationship to be built up between students and teachers, which was seen as conducive to the development of a doctor’s own identity.

The relatively small number of participants in relation to the size of the semester is offset by the extremely positive response of the participating students, which encourages further development and dissemination of the curriculum.

An adaptation for postgraduate medical education is also conceivable in the future.

## Acknowledgements

We would like to thank all – including former – members of the “LongProf” working group, the participating students for their openness and curiosity, the Study Dean’s Office of Jena University Hospital for their organisational support, Prof. Dr. Jutta Bleidorn for the support from the Institute of General Medicine, Dr. Carolin Klingner, Dr. Anne Klemm, Prof. Dr. Ulrich Wedding, Dr. Ulf Zitterbart, and Dr. Eckart Zillessen for their participation in the kickoff weekend, Prof. Dr. Andrea Geissler, Dr. Paula Linden, and Oskar Masztalerz for delivering external lectures, as well as Gesine Müller for assistance with data analysis. Exchanges with colleagues from other medical schools, including Augsburg, Witten/Herdecke, and the Technical University of Munich, were also very fruitful.

## Notes

### Funding

This work was supported by the Strategy and Innovation Funds of the Federal State of Thuringia for projects of the University Hospital Jena from chapter 07 50 68202.

### Author’s ORCID

Konrad Schmidt: [0000-0001-5879-0664]

## Competing interests

The authors declare that they have no competing interests. 

## Supplementary Material

Learning objectives and didactic methods of the individual events of the LongProf curriculum

Focus group interview guide

Quantitative evaluation: Individual items for the evaluation of events and teachers

## Figures and Tables

**Table 1:**
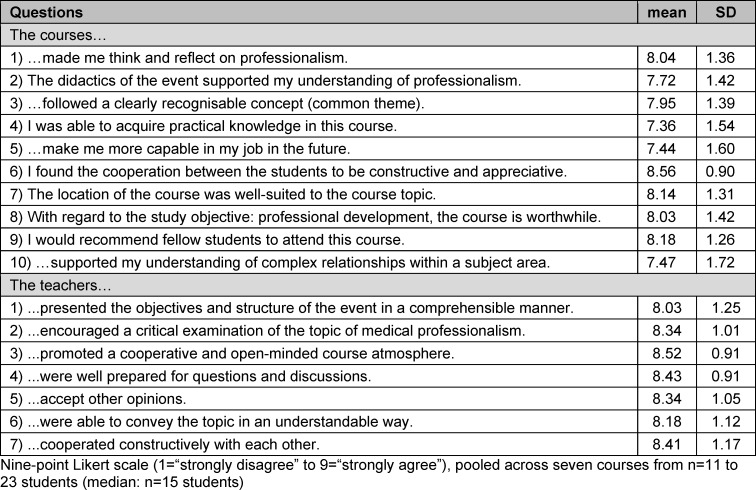
Quantitative evaluation: single items for evaluating the course and teachers

**Table 2:**
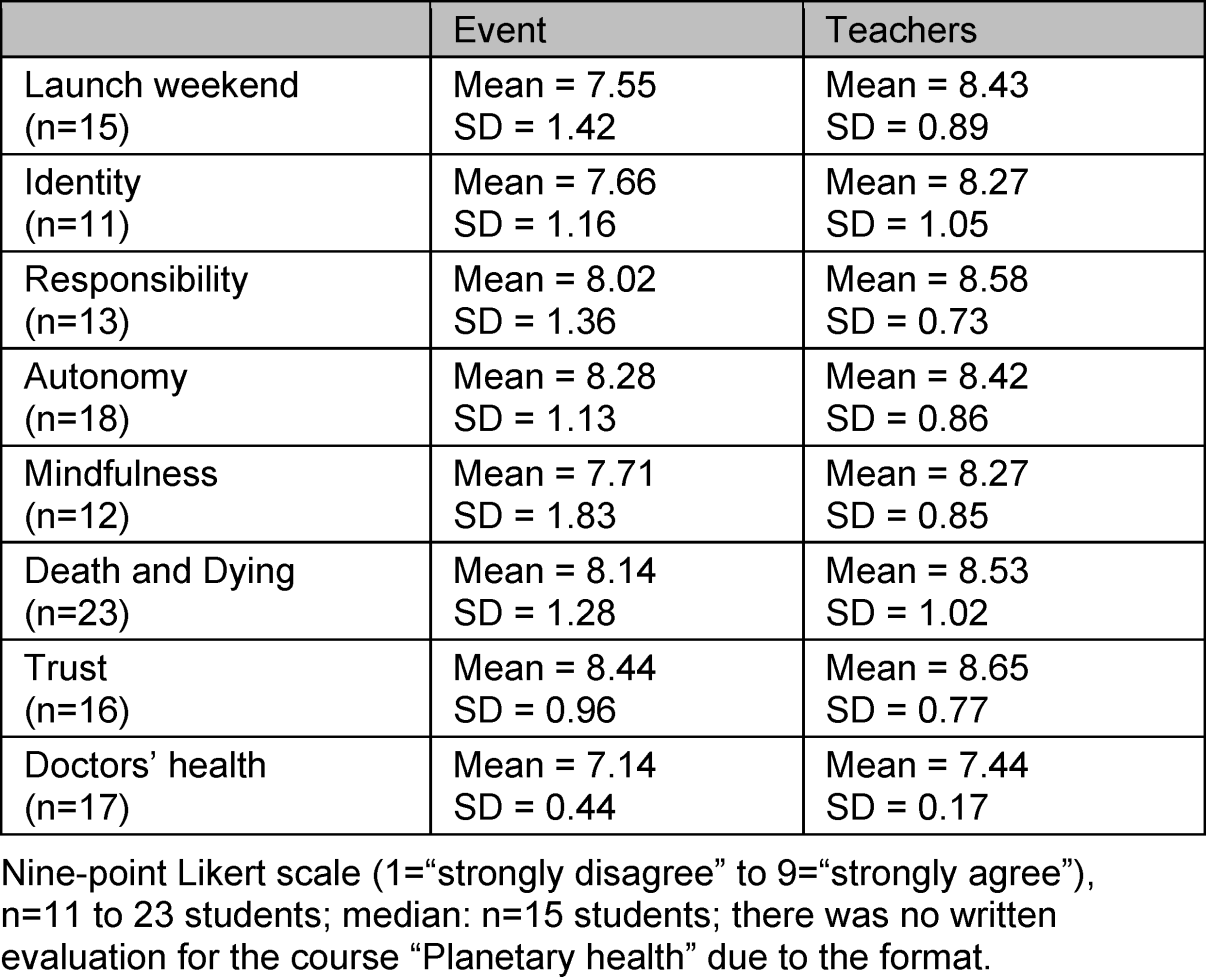
Quantitative evaluation, summative assessment of eight courses

**Table 3:**
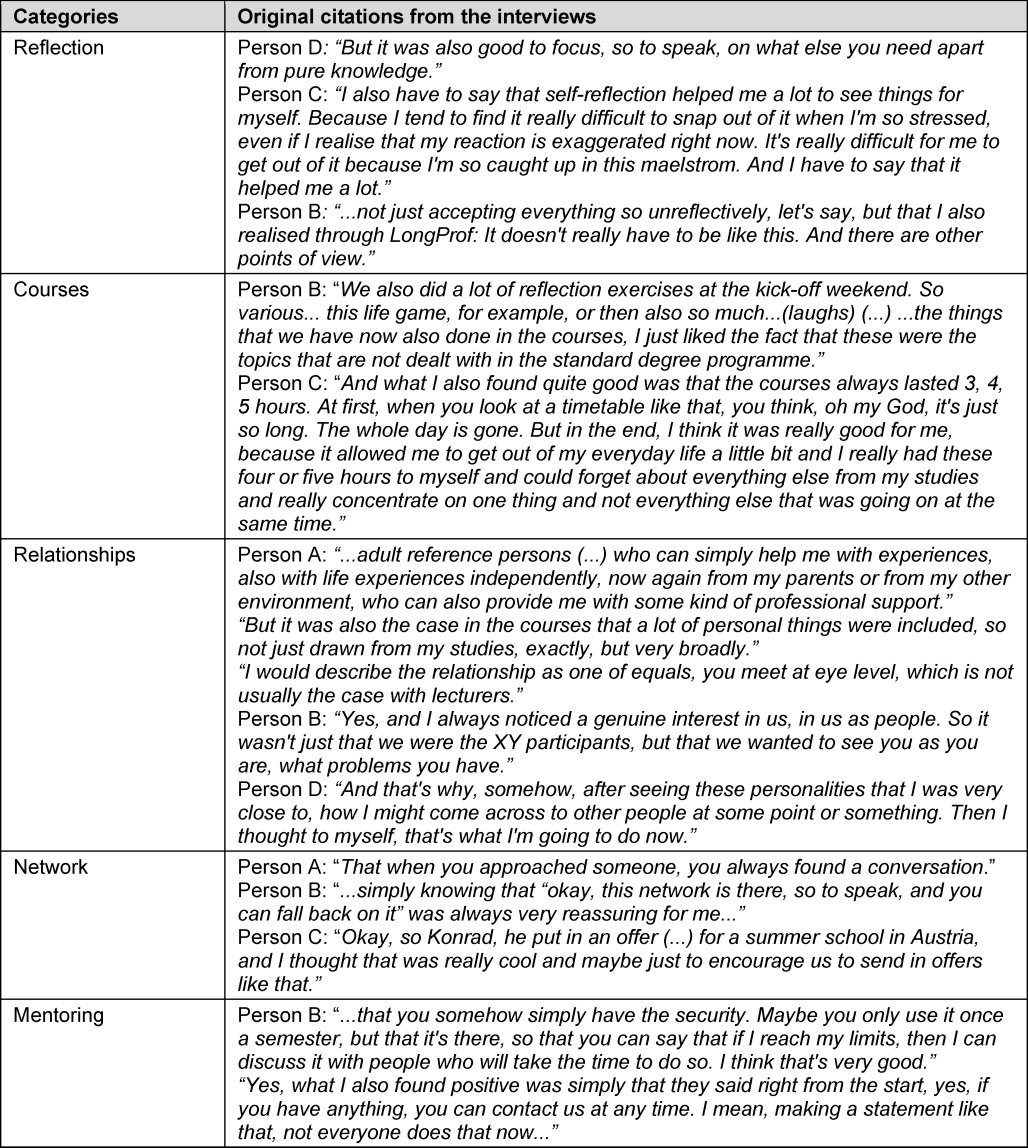
Citations from the group interview, end of winter semester 2021/22
