# Supplementary material for: Professional development of medical students – piloting a longitudinal curriculum at Jena University Hospital (LongProf)
Source: GMS J Med Educ. 2024 Sep 16;41(4):Doc44. doi: 10.3205/zma001699 (PMC11474655; doi:10.3205/zma001699)
Supplement: Learning objectives and didactic methods of the individual events of the LongProf curriculum [file JME-41-44-s-001.pdf]

Attachment 1: Learning objectives and didactic methods of the individual events of the LongProf curriculum

**Topic**<br>***(Venue)***	**Learning objectives**	**Corresponding competence of the NKLM 2.0**	**Methods**	**Volume**
**Launch weekend**<br>(*external venue, rural hostel)*	o formation of a group identity<br>o identify own ideas and expectations of medical professionalism<br>o reflect on their own biography with regard to their choice of medical profession<br>o getting to know medical role models	VIII.6-03.1<br>*...they are capable of self-knowledge, self-reflection, self-criticism and self-development.*	o multimodal mindfulness exercises at the start of the day (*Taiji*, “photo walk”, meditation)<br>o walk alone in silence with the question “What makes a good doctor for me?”<br>o Lecture: Frameworks of medical professionalism/situational model as a framework concept<br>o writing discussion on topics of medical professionalism<br>o Representation of typical medical professional constellations as a still image<br>o biography work as a board game<br>o “me in 10 years” - visualisation of the future biography with pipe cleaners<br>o **world cafe with medical colleagues from different specialties**, survey on individual career paths<br>o self-reflection/exercise: “Letter to myself”	26 Teaching Units (TU)
**Identity**<br>(*course room of the University of Jena*)	o know the basic features of different personality typologies<br>o reflect on the limits, strengths and weaknesses of their own personality<br>o transfer the consequences of this reflection to their own medical work<br>o name their own medical role models and guiding principles		o lecture: Geneva Declaration [29] and Physician Charter according to the American Board of Internal Medicine [6]<br>o collection and presentation of own medical role models and guiding principles<br>o Lecture: Personality typology according to Riemann-Thomann [30]<br>o exercise: Self-location of own personality visualised in a spatial constellation<br>o group discussion on the effects of personality traits on clinical work	6 TU
**Responsibility**<br>(*course room of the University of Jena*)	o reflect on the experience of responsibility in a medical context<br>o promote the ability to assume responsibility<br>o experience self-efficacy in an example	VIII.6-03.1.2 *…they can realistically assess their own skills and align the assumption of tasks and responsibilities accordingly*.	o partner exercise “blind man's walk”<br>o case vignettes from different situational levels as a constellation in the room<br>o Lecture: Legal and moral aspects of responsibility in a medical context<br>o report from a young professional on dealing with difficult situations	6 TU

Attachment 1 to Schmidt K, Siller K, Rißmann J, Andlauer M, Feustel J, Klein F, Petruschke I, Wagner L, Schulz S. *Professional development of medical students – piloting a longitudinal curriculum at Jena University Hospital (LongProf)*. GMS J Med Educ. 2024;41(4):Doc44. DOI: 10.3205/zma001699

**Topic** ***(Venue)***	**Learning objectives**	**Corresponding competence of the NKLM 2.0**	**Methods**	**Volume**
**Autonomy** *(circus tent)*	○ perceive their own autonomy, freedom and creative possibilities in everyday medical and student life<br>○ be able to classify first and second order autonomy [18], self-determination theory according to Deci and Ryan [23]	VIII.6-04.2.3 *…they can explain the ethical and legal foundations of the principle of patient autonomy.*	○ experience of autonomy: Painting with a handicap<br>○ Lecture: First and second order autonomy [31], self-determination theory according to Deci and Ryan [32]<br>○ Reflection: How does the autonomy of a doctor differ from that of a circus artist?<br>○ representation of autonomy conflicts as improvisational theatre	6 TU
**Mindfulness** *(Dornburger Castle)*	○ identify thought patterns and beliefs in themselves and their impact on patient contact<br>○ apply techniques for distancing oneself from negative thought patterns<br>○ know how mindfulness can be used in a therapeutic context and for your own resilience	VIII.6-03.2.2 *…they can use individual strategies to cope with and reduce stress and explain appropriate professional support services.*	○ unintentional walk as a mindfulness exercise<br>○ guided mindfulness meditation and body awareness<br>○ **Lecture open to the public (Prof. Angela Geissler)**: **Origin and evidence of clinical application of mindfulness-based practices**<br>○ role play for identifying and distancing thought patterns in patient contact	6 TU
**Death and passing** *(church devotional room)*	○ explore own experiences, emotions and cognitions about death and dying<br>○ reflect on the meaning and limits of the medical role in dealing with death and dying<br>○ realise their own finiteness<br>○ address and reduce fear of dealing with death in a clinical context	11.4.3.4 *…they can explain the cultural and historical conditionality of medicine and its influence on medical training.*<br>14c.3.2.11 *…they can communicate truthfully and empathically with the dying and their relatives.*<br>18.3.4.1 *…they are able to reflect on the cultural and temporal nature and ideological differences in dealing with dying and death.*	○ small group discussion about own experiences with death and dying, significance for own world view and the doctor as “opponent of death”<br>○ Lecture: Ethical and legal aspects of dealing with death, withdrawal of treatment and euthanasia<br>○ list of the students' own case vignettes<br>○ multimodal offers for individual asynchronous consolidation (including writing and drawing exercises, film and book recommendations as well as follow-up with teachers in attendance)	6 TU
**Trust** *(course room of the University of Jena)*	○ be able to evaluate trust in the medical profession for themselves and for the doctor-patient relationship<br>○ reflect on the importance of honesty and safety, but also vulnerability in patients and practitioners<br>○ know aspects of the formation, loss and restoration of trust	VIII.6-04.3, VIII.6-04.3.1 *…they know and take into account the importance and responsible handling of trust in doctor-patient relationships. They can responsibly apply measures to build trust.*	○ group constellation on questions of trust<br>○ group reflection on the question “What is needed to build trust from the patient or doctor's perspective?”<br>○ Lecture: Origins and definitions of personal trust and trust in the healthcare system<br>○ experience report from a patient followed by a Q&A session<br>○ role play in small groups based on case vignettes<br>○ **Lecture open to the public “Trust in medicine” (Dr. Paula Linden)**	6 TU

Topic<br>*(Venue)*	Learning objectives	Corresponding competence of the NKLM 2.0	Methods	Volume
**How do I stay healthy as a doctor?**<br>*(course room of the University of Jena)*	○ get to know the concept of salutogenesis<br>○ recognising the importance of one's own moral values as an aspect of health<br>○ becoming aware<br>- of the special professional demands of being a doctor<br>- of own resources at micro, meso and macro level	*VIII.6-03.2.2 …they can use individual strategies to cope with and reduce stress and explain appropriate professional support services.*	○ group and partner discussion on own ideas of personal health and the special requirements of the medical profession<br>○ reflection on own moral values, individual work with “value target”<br>○ theory-based presentation on salutogenesis and concepts of health<br>○ practical exercises: *Taiji* and *Qigong*	6 TU
**Planetary health**<br>*(living room, forest, lecture hall)*	○ understand health as a supra-individual concept<br>○ understand systemic interactions between economic, ecological, political and cultural determinants using the example of climate change<br>○ placing one's own clinical activity in a global context, exploring one's own potential for change	*VIII.4-19.1.1.new.3 and 4 ...they can name the terms, models and variables of public health and global health and name health policy goals and challenges at national and international level.*	○ **Lecture open to the public: “Planetary health - challenge of the 21st century” (Oscar Masztalerz,** *Charité, School of Public Health Berlin, German Advisory Council on Global Change*)<br>○ teaching format developed together with students, consisting of three thematic regulars' tables, intensive nature experience and reflection	6 TU

(The involvement of external speakers is highlighted)
