# Supplementary material for: Professional development of medical students – piloting a longitudinal curriculum at Jena University Hospital (LongProf)
Source: GMS J Med Educ. 2024 Sep 16;41(4):Doc44. doi: 10.3205/zma001699 (PMC11474655; doi:10.3205/zma001699)
Supplement: Focus group interview guide [file JME-41-44-s-002.pdf]

Attachment 2: Focus group interview guide

Goals:

- Evaluate the impact of the LongProf intervention on students, differentiated by content, teaching formats and personnel
- Generate impulses for the continuation of LongProf

Categories:

1. ***Impact of the LongProf intervention***

When you look back on the last semester and in particular on the LongProf events, what has touched you? (What has LongProf changed for you in your life as a medical student?)

- *How does LongProf help you? How does LongProf help you to develop your professionalism? How do you recognise the development?*
- *Do you see/have conflicts/tensions within LongProf? Were some exercises too challenging?*
- *What wishes are still open?*
- *What do you expect to have changed after completing LongProf? Personal/structural?*

2. ***Relationships***

One of the special features of LongProf is the composition of both teachers and students (semesters). How do you perceive the relationships in the group?

- *What role does this composition play for you - advantages and disadvantages? (professions, age)*
- *How do you perceive the relationships in the group? (fellow students with each other, teachers with each other, students and teacher, continuity, own choice in tandem)*
- *What do you wish for from the relationships? What was helpful?*

3. ***Organisational framework***

How do you see LongProf in the long term? What is important to you for the future?

- *What was the significance of the launch weekend for you?*
- *Assuming that LongProf is made permanent, what would you wish for differently?*
- *How did you find the mentoring offer and the impulses by e-mail?*

Attachment 2 to Schmidt K, Siller K, Rißmann J, Andlauer M, Feustel J, Klein F, Petruschke I, Wagner L, Schulz S. *Professional development of medical students – piloting a longitudinal curriculum at Jena University Hospital (LongProf)*. GMS J Med Educ. 2024;41(4):Doc44. DOI: 10.3205/zma001699
