# Supplementary material for: Professional development of medical students – piloting a longitudinal curriculum at Jena University Hospital (LongProf)
Source: GMS J Med Educ. 2024 Sep 16;41(4):Doc44. doi: 10.3205/zma001699 (PMC11474655; doi:10.3205/zma001699)
Supplement: Quantitative evaluation: Individual items for the evaluation of events and teachers [file JME-41-44-s-003.pdf]

Attachment 3: Quantitative evaluation: Individual items for the evaluation of events and teachers

Questions	Launch-weekend<br>*n* = 15	Identity<br>*n* = 11	Responsi-bility<br>*n* = 13	Autonomy<br>*n* = 18	Mindfulnes<br>*n* = 12	Death and dying<br>*n* = 23	Trust<br>*n* = 16	Doctor's health<br>n=17
The event…								
1) …made me think and reflect on professionalism.	*Mean* = 8.00<br>*SD* = 1.63	*Mean* = 7.73<br>*SD* = .86	*Mean* = 8.54<br>*SD* = .50	*Mean* = 8.50<br>*SD* = .69	*Mean* = 7.75<br>*SD* = 1.69	*Mean* = 8.04<br>*SD* = 1.40	*Mean* = 8.33<br>*SD* = 1.19	Mean = 7.35<br>SD = 1.77
2) The didactics of the event supported my understanding of professionalism.	*Mean* = 7.47<br>*SD* = 1.54	*Mean* = 7.18<br>*SD* = 1.11	*Mean* = 8.00<br>*SD* = .96	*Mean* = 8.11<br>*SD* = .99	*Mean* = 7.33<br>*SD* = 1.89	*Mean* = 7.87<br>*SD* = 1.36	*Mean* = 8.38<br>*SD* = .93	Mean = 7.12<br>SD =1.80
3) …followed a clearly recognizable concept (red thread).	*Mean* = 7.53<br>*SD* = 1.50	*Mean* = 7.64<br>*SD* = .98	*Mean* = 8.15<br>*SD* = 1.17	*Mean* = 8.50<br>*SD* = 1.01	*Mean* = 7.58<br>*SD* = 1.89	*Mean* = 8.22<br>*SD* = 1.14	*Mean* = 8.47<br>*SD* = .88	Mean = 7.24<br>SD =1.71
4) I was able to acquire practical knowledge in this course.	*Mean* = 6.47<br>*SD* = 1.41	*Mean* = 6.80<br>*SD* = 0.87	*Mean* = 7.38<br>*SD* = 1.08	*Mean* = 7.83<br>*SD* = 1.17	*Mean* = 7.08<br>*SD* = 2.25	*Mean* = 7.74<br>*SD* = 1.33	*Mean* = 8.53<br>*SD* = .62	Mean = 6.65<br>SD =1.80
5) …makes me more capable in my job in the future.	*Mean* = 6.47<br>*SD* = 1.54	*Mean* = 7.09<br>*SD* = 1.08	*Mean* = 7.54<br>*SD* = 1.78	*Mean* = 8.06<br>*SD* = 1.43	*Mean* = 7.33<br>*SD* = 1.89	*Mean* = 7.96<br>*SD* = 1.30	*Mean* = 8.33<br>*SD* = .79	Mean = 6.41<br>SD =1.58
6) I found the cooperation between the students to be constructive and appreciative.	*Mean* = 8.40<br>*SD* = 1.20	*Mean* = 8.27<br>*SD* = .75	*Mean* = 8.77<br>*SD* = .58	*Mean* = 8.78<br>*SD* = .53	*Mean* = 8.75<br>*SD* = .60	*Mean* = 8.83<br>*SD* = .48	*Mean* = 8.80<br>*SD* = .54	Mean = 7.82<br>SD =1.47
7) The location of the course was well suited to the course topic.	*Mean* = 8.20<br>*SD* = 1.28	*Mean* = 8.45<br>*SD* = .78	*Mean* = 8.23<br>*SD* = 1.62	*Mean* = 8.06<br>*SD* = 1.35	*Mean* = 8.75<br>*SD* = .60	*Mean* = 8.35<br>*SD* = .96	*Mean* = 7.93<br>*SD* = 1.48	Mean = 7.41<br>SD =1.58
8) With regard to the study objective: professional development, the course is worthwhile.	*Mean* = 7.87<br>*SD* = 1.41	*Mean* = 7.73<br>*SD* = 1.05	*Mean* = 8.38<br>*SD* = .62	*Mean* = 8.61<br>*SD* = .89	*Mean* = 7.58<br>*SD* = 1.98	*Mean* = 8.09<br>*SD* = 1.47	*Mean* = 8.67<br>*SD* = .60	Mean = 7.18<br>SD =1.85
9) I would recommend fellow students to attend this course.	*Mean* = 8.13<br>*SD* = 1.36	*Mean* = 8.55<br>*SD* = .66	*Mean* = 8.08<br>*SD* = 1.44	*Mean* = 8.39<br>*SD* = 1.06	*Mean* = 7.58<br>*SD* = 1.55	*Mean* = 8.39<br>*SD* = 1.05	*Mean* = 8.67<br>*SD* = .60	Mean = 7.53<br>SD =1.55
10) ...supported my understanding of complex relationships within a subject area.	*Mean* = 6.93<br>*SD* = 1.34	*Mean* = 7.09<br>*SD* = 1.56	*Mean* = 7.08<br>*SD* = 1.94	*Mean* = 8.00<br>*SD* = 1.37	*Mean* = 7.33<br>*SD* = 2.21	*Mean* = 7.91<br>*SD* = 1.59	*Mean* = 8.33<br>*SD* = 1.19	Mean = 6.65<br>SD =1.73
The teachers…								
1) …resented the objectives and structure of the event in a comprehensible manner.	*Mean* = 7.73<br>*SD* = 1.29	*Mean* = 7.64<br>*SD* = 1.23	*Mean* = 8.38<br>*SD* = 1.15	*Mean* = 8.17<br>*SD* = .83	*Mean* = 7.92<br>*SD* = 1.04	*Mean* = 8.35<br>*SD* = 1.05	*Mean* = 8.47<br>*SD* = .72	Mean = 7.41<br>SD = 1.91
2) …encouraged a critical examination of the topic of medical professionalism.	*Mean* = 8.53<br>*SD* = .72	*Mean* = 8.09<br>*SD* = 1.16	*Mean* = 8.38<br>*SD* = .84	*Mean* = 8.67<br>*SD* = .58	*Mean* = 8.50<br>*SD* = .76	*Mean* = 8.57<br>*SD* = .71	*Mean* = 8.53<br>*SD* = .96	Mean = 7.35<br>SD = 1.46
3) …promoted a cooperative and open-minded course atmosphere.	*Mean* = 8.80<br>*SD* = .54	*Mean* = 8.45<br>*SD* = .89	*Mean* = 8.77<br>*SD* = .58	*Mean* = 8.67<br>*SD* = .67	*Mean* = 8.42<br>*SD* = .76	*Mean* = 8.74<br>*SD* = .53	*Mean* = 8.73<br>*SD* = .68	Mean = 7.59<br>SD = 1.54
4) …were well prepared for questions and discussions.	*Mean* = 8.60<br>*SD* = .61	*Mean* = 8.36<br>*SD* = .98	*Mean* = 8.62<br>*SD* = .49	*Mean* = 8.50<br>*SD* = .76	*Mean* = 8.00<br>*SD* = .82	*Mean* = 8.74<br>*SD* = .53	*Mean* = 8.87<br>*SD* = .50	Mean = 7.65<br>SD = 1.50
5) …accepted dissenting opinions.	*Mean* = 8.67<br>*SD* = 0.79	*Mean* = 8.45<br>*SD* = .78	*Mean* = 8.77<br>*SD* = .42	*Mean* = 8.28<br>*SD* = .99	*Mean* = 8.33<br>*SD* = .85	*Mean* = 8.45<br>*SD* = .99	*Mean* = 8.47<br>*SD* = 1.09	Mean = 7.47<br>SD = 1.42
6) …were able to convey the topic in an understandable way.	*Mean* = 8.00<br>*SD* = .82	*Mean* = 8.18<br>*SD* = 1.19	*Mean* = 8.54<br>*SD* = .63	*Mean* = 8.22<br>*SD* = 1.08	*Mean* = 8.25<br>*SD* = .83	*Mean* = 8.57<br>*SD* = .82	*Mean* = 8.60<br>*SD* = .71	Mean = 7.12<br>SD = 1.65
7) …cooperated constructively with each other.	*Mean* = 8.67<br>*SD* = .70	*Mean* = 8.73<br>*SD* = .62	*Mean* = 8.62<br>*SD* = .62	*Mean* = 8.44<br>*SD* = .83	*Mean* = 8.50<br>*SD* = .65	*Mean* = 8.30<br>*SD* = 1.80	*Mean* = 8.87<br>*SD* = .34	Mean = 7.47<br>SD = 1.50

Nine-point Likert scale for all eight courses from n=11-23 students (median: n=15 students)

Attachment 3 to Schmidt K, Siller K, Rißmann J, Andlauer M, Feustel J, Klein F, Petruschke I, Wagner L, Schulz S. *Professional development of medical students – piloting a longitudinal curriculum at Jena University Hospital (LongProf)*. GMS J Med Educ. 2024;41(4):Doc44. DOI: 10.3205/zma001699
